# Patients with vitiligo have a distinct affective temperament profile: A cross‐sectional study using Temperament Evaluation of Memphis, Paris, and San Diego Auto‐Questionnaire

**DOI:** 10.1002/ski2.157

**Published:** 2022-08-12

**Authors:** Noureddine Litaiem, Olfa Charfi, Sami Ouanes, Soumaya Gara, Faten Zeglaoui

**Affiliations:** ^1^ Department of Dermatology Charles Nicolle Hospital Tunis Tunisia; ^2^ Faculty of Medicine of Tunis University of Tunis el Manar Tunis Tunisia; ^3^ Department of Psychiatry Hamad Medical Corporation Doha Qatar

## Abstract

**Background:**

Vitiligo is a skin disease associated with significant psychiatric comorbidities. Affective temperaments represent the inherited foundation of personality and represent the biologically stable part of emotional reactivity. Studies assessing the temperament profile of patients with vitiligo are still scarce.

**Method:**

This was a cross‐sectional study enrolling 34 patients with vitiligo and 34 age and sex‐matched healthy controls. Affective temperament profile was investigated using the Temperament Evaluation of Memphis, Paris, and San Diego Auto‐questionnaire. Dermatology life quality index was used to evaluate disease impact on patients' quality of life.

**Results:**

We found significant differences with vitiligo patients scoring higher in anxious (11.5 ± 4.76 vs. 9.06 ± 6.22; *p* = 0.036) and lower in hyperthymic (5.79 ± 3.82 vs. 7.5 ± 3.64; *p* = 0.027) temperaments. Vitiligo worsening reported by patients was associated with recent psychological stressors in 18 cases (52.9%) and Koebner phenomenon in 20 cases (58.8%). Koebner phenomenon was more frequently associated with the hyperthymic temperament (*p* = 0.035). Cyclothymic temperament was positively correlated with Dermatology life quality index (rho = 0.417, *p* = 0.014).

**Conclusions:**

This study demonstrated that patients with vitiligo have a distinct premorbid temperament profile. Having low hyperthymic and high anxious temperament traits seem to predispose patients to be less resilient to psychological stressors. A better understanding of the affective traits of vitiligo patients would be vital in personalising and adapting the management of this chronic skin disease.

## Graphical abstract

This study demonstrated that patients with vitiligo have a distinct temperament profile. Having low hyperthymic and high anxious temperament traits seem to predispose patients to be less resilient to psychological stressors.

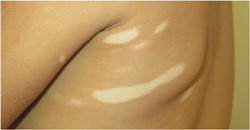

1

**What is already known about this topic?**

- Temperament refers to the genetically inherited and relatively stable ‘core’ of personality that appears early in life.
- Both affective temperament and vitiligo share genetic backgrounds.

**What does this study add?**

- Patients with vitiligo have a distinct premorbid temperament profile.
- Having low hyperthymic and high anxious temperament traits seem to predispose vitiligo patients to be less resilient to psychological stressors.

## 2 INTRODUCTION

Temperament refers to the genetically inherited and relatively stable ‘core’ of personality that appears early in life^1^, ^2^, ^3^ and represents the emotional and affective basis of the individual.^4^

Definitions of temperament have evolved in the past century and temperament has been gradually separated from the concept of personality.^5^ In 1921, Kraepelin describes four basic affective dispositions namely depressive, cyclothymic, irritable, and maniac which he believed to be the precursors of affective psychoses.^6^ More recently, Akiskal et al. have defined the existence of five affective temperaments (depressive, cyclothymic, hyperthymic, irritable, anxious) which predispose to the onset of a thymic disorder.^7^ Hyperthymic patients tend to have high levels of energy, enthusiasm and positivity. Cyclothymic patients tend to experience mood shifts and emotional ups and downs. These affective temperaments could be permanent premorbid traits or prodromal expressions of mood disorders.^8^

Several studies also support that affective temperaments could serve as protective or risk factors of some medical conditions linked to psychological factors or determinants.^8^ For instance, the hyperthymic temperament was associated with increased Brain‐Derived Neurotrophic Factor serum levels in patients with hypertension (but not in controls), suggesting that the hyperthymic temperament may exert a protective role on peripheral neurons and vascular cells in patients with hypertension.^9^ Similarly, patients with Crohn's disease had significantly higher scores of depressive, cyclothymic, and anxious temperaments than controls.^10^ In these patients, disease severity was positively associated with depressive, cyclothymic, and anxiety subscales of Temperament Evaluation of Memphis, Pisa, Paris, and San Diego auto‐questionnaire (TEMPS‐A). Likewise, patients with chronic idiopathic urticaria had higher scores of novelty seeking and lower scores of cooperativeness, reward dependence, and self‐directedness than controls.^11^

Skin diseases, in particular, show tight links with psychiatric disorders, self‐esteem, and personality. Vitiligo is an autoimmune skin disease characterised by selective loss of melanocytes and acquired depigmentation and is associated with significant psychiatric comorbidities including depression and anxiety.^12^, ^13^, ^14^ Up to 75% of patients with vitiligo have a co‐occurring psychiatric disorder.^15^, ^16^

Furthermore, vitiligo can be a source of social stigma, thus affecting psychological wellbeing. Vitiligo has been associated with low self‐esteem, depression, and suicidality; all of which leading to an overall poor prognosis in particular with regards to the quality of life.^17^ Since temperament might ‘modulate’ one's self‐esteem, and propensity to depression and anxiety, temperament might be a prognostic factor in patients with vitiligo.^18^, ^19^ Psychological stressors are also known potential triggers for vitiligo, and since temperament modulates the way we cope with the psychological stressors, some temperaments might be risk factors for vitiligo.^17^

Contrasting with the relative abundance of studies about the prevalence of psychiatric disorders in patients with vitiligo, studies assessing the temperament profile of these patients are still scarce. To the best of our knowledge, only three studies have investigated the temperament traits in vitiligo patients.^20^, ^21^, ^22^ None of these studies used TEMPS‐A.

Our study aimed to investigate the affective temperament profile of patients with vitiligo using TEMPS‐A and to examine the associations between temperament types and clinical features.

## 3 METHODS

### 3.1 Study design

This was a cross‐sectional study conducted at the Department of Dermatology, Charles Nicolle Hospital, Tunis, Tunisia between October 2018 and June 2019.

Thirty‐four patients with vitiligo and 34 age and sex‐matched healthy controls were enroled. The controls didn't have any form of skin disease, auto immune disease nor psychiatric disease.

The diagnosis of vitiligo was made based on clinical findings and the Wood lamp examination.

Patients who reported a history of any psychiatric disorder, who were diagnosed with another dermatological disease, who were unable to read and understand the questions, or who were younger than 21 years, as well as pregnant women, were not included in the study. All patients gave informed consent and agreed to participate in the study.

Two dermatologists (Dr. Olfa Charfi and Dr. Noureddine Litaiem) collected information on clinical features of vitiligo including localization of the lesions (face, arms, external genital organs), vitiligo type, and disease course. Two disease triggers or aggravating factors were considered: Koebner phenomenon and recent psychological stressor (happening in the past 6 months preceding disease worsening). Koebner phenomenon refers to a form of isomorphic response characterised by the appearance of disease‐related skin lesions after trauma.^23^ Affective temperaments were assessed using TEMPS‐A. The impact of vitiligo on patient's quality of life was evaluated using the Dermatology Life Quality Index (DLQI).

### 3.2 Instruments

* TEMPS‐A The affective temperament profile of all included subjects was investigated using the TEMPS‐A.TEMPS‐A is an auto‐questionnaire developed by Akiskal *et al.*, used to assess the depressive, cyclothymic, hyperthymic, irritable, and anxious temperaments.^7^

The external validity of TEMPS‐A against Temperament and Character Inventory (TCI)^24^ was established.^2^ TEMPS‐A is the most widely used instrument to evaluate affective temperaments.^7^, ^25^ It was translated into at least 25 languages and validated in more than 10 languages including Arabic.^2^, ^26^ The TEMPS‐A consists of 109 (for women) or 110 (for men) ‘yes’ or ‘no’ questions. The questionnaire is designed to assess the five predominant affective temperaments (depressive: 21 items, cyclothymic: 21 items, hyperthymic: 21 items, irritable: 21 items and anxious: 26 items).

The validated Arabic version of the questionnaire was used. This version proved to be appropriate in assessing temperament profile in Tunisian patients with skin diseases.^27^

* DLQI: The impact of vitiligo on patient's quality of life was measured using the Arabic version of DLQI. Dermatology life quality index is a 10‐item self‐questionnaire comprising 10 questions. Each question is rated from zero to three for a total score ranging between zero (no impact on the quality of life) and 30 (maximum impact on the quality of life). It assesses the patient's perception of the impact of the illness on various aspects (symptoms, daily activities, leisure, work, or studies…) and thus during the previous 7 days.

### 3.3 Statistics

Data of all included patients were analysed using SPSS version 19 (SPSS Inc.). Continuous variables were expressed by means and standard deviation. Shapiro‐Wilk test was used to assess the normality distribution of the variables. To compare categorical variables between patients and controls, we used Pearson's Chi‐square, and in case of non‐validity, Fisher's exact test. To compare continuous variables between groups, we used the *T*‐test in case of normality, and Mann–Whitney *U* test in case of non‐normality. Spearman's non‐parametric correlations were used to examine associations between age, duration of the disease, DLQI, and temperament scores. A *p*‐value of less than 0.05 was considered significant.

## 4 RESULTS

Thirty‐four^28^ vitiligo patients and 34 healthy controls were enroled in the study. The mean age of patients with vitiligo was 51 ± 14 years. There were 24 female and 10 male patients in the vitiligo group. Since groups were age and gender‐matched, there were no significant differences, with regards to age and gender between patients and controls.

**FIGURE 1:**
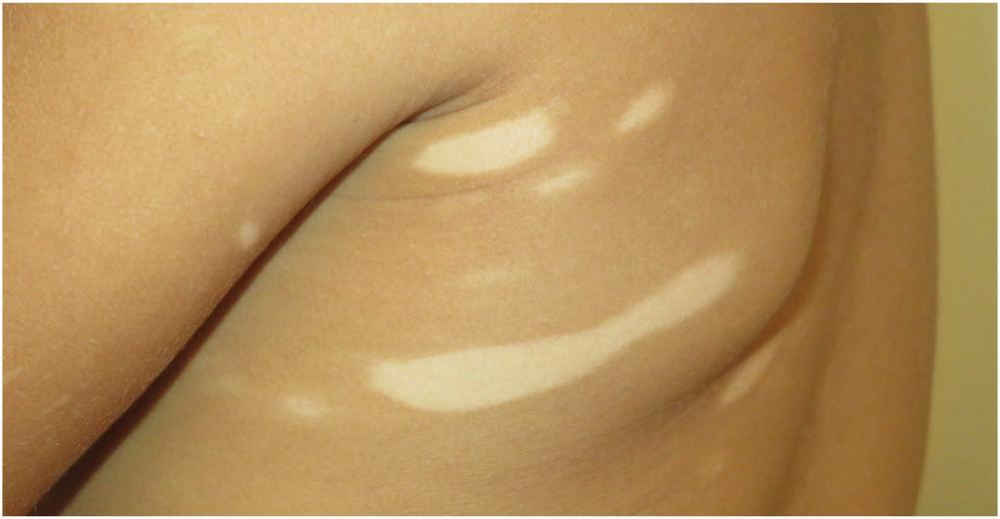
Segmental vitiligo

**FIGURE 2:**
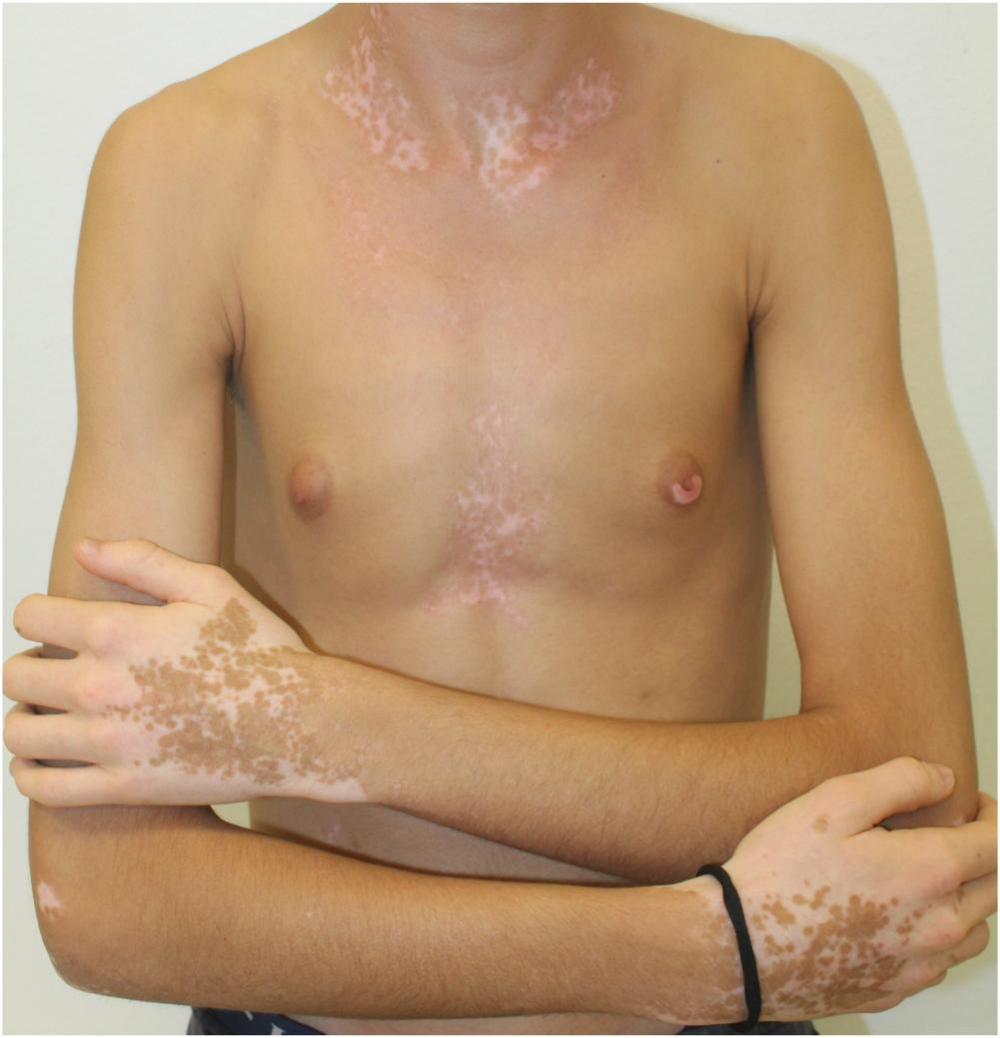
Nonsegmental vitiligo

In the vitiligo group, the mean disease duration was 10 ± 12 years (extremes: 1–42 years). There were seven cases of segmental vitiligo (21%) (Figure 1), 24 cases of non‐segmental vitiligo (70%) (Figure 2) and three cases of mixed vitiligo (9%). Head and neck, hand and external genital organ involvement were found in 25 (73.5%), 26 (76.5%) and 12 (35.3%) cases respectively. Disease worsening was associated with recent psychological stressors in 18 cases (52.9%) and Koebner phenomenon in 20 cases (58.8%). The mean DLQI score in vitiligo patients was 5.88 ± 4.71 (extremes: 1–20).

Affective temperament scores are summarised in Table 1. Anxious temperament scores were significantly higher in vitiligo patients (11.5 ± 4.76 vs. 9.06 ± 6.22; *p* = 0.036) while hyperthymic temperament scores were significantly higher in the control group (5.79 ± 3.82 vs. 7.5 ± 3.64; *p* = 0.027). Both groups scored highest in anxious followed by depressive temperament subscales (Table 1).

**TABLE 1:** Comparison of temperament sores among vitiligo patients and healthy controls

	Groups	N	Mean ± SD	*p*
Depressive	Vitiligo	34	10.47 ± 4.4	0.119
	Controls	34	8.76 ± 3.93	
Cyclothymic	Vitiligo	34	6.97 ± 4.07	0.739
	Controls	34	6.41 ± 3.32	
Hyperthymic	Vitiligo	34	5.79 ± 3.82	**0.027**
	Controls	34	7.5 ± 3.64	
Irritable	Vitiligo	34	5.94 ± 3.57	0.169
	Controls	34	4.73 ± 3.02	
Anxious	Vitiligo	34	11.5 ± 4.76	**0.036**
	Controls	34	9.06 ± 6.22	

*Note*: Bold values: the score is significantly higher in subgroup analysis.

Abbreviation: SD, standard deviation.

In the vitiligo group, anxious and depressive temperament scores were significantly higher in females (Table 2). There was no significant association between affective temperament, vitiligo subtypes, psychological stress, or disease duration. Koebner phenomenon was more frequently associated with hyperthymic temperament (*p* = 0.035).

**TABLE 2:** comparison of temperament scores according to age, gender, and clinical features of vitiligo

		Depressive	Cyclothymic	Hyperthymic	Irritable	Anxious
Gender	F	11.58 ± 4.60*	7.54 ± 4.28	5.67 ± 4.33	5.79 ± 3.30	12.79 ± 4.90*
	M	7.80 ± 2.44	5.60 ± 3.34	6.10 ± 2.33	6.30 ± 4.35	8.40 ± 2.59
Age	Less than 50 years	10.23 ± 4.49	8.08 ± 4.23	5.46 ± 3.10	6.69 ± 4.05	12.85 ± 5.26
	Higher than 50 years	10.62 ± 4.46	6.29 ± 3.93	6.00 ± 4.27	5.48 ± 3.27	10.67 ± 4.35
Duration of disease	Less than 5 years	10.53 ± 4.72	7.47 ± 3.91	5.65 ± 3.48	6.88 ± 3.60	12.12 ± 4.61
	Higher than 5 years	9.94 ± 3.86	6.38 ± 4.43	6.13 ± 4.30	4.75 ± 3.34	10.44 ± 4.77
Forms of vitiligo	SV	9.29 ± 4.11	7.00 ± 2.89	4.71 ± 3.55	8.57 ± 3.82	11.57 ± 4.54
	NSV	11.52 ± 4.45	7.48 ± 4.54	6.26 ± 4.20	5.61 ± 3.30	11.83 ± 5.00
Facial involvment	Yes	10.76 ± 4.25	7.72 ± 4.26	5.60 ± 3.34	6.04 ± 3.75	11.76 ± 4.16
	No	9.67 ± 5.00	4.89 ± 2.76	6.33 ± 5.12	5.67 ± 3.24	10.78 ± 6.40
Hand involvement	Yes	9.69 ± 4.36	7.00 ± 4.52	6.50 ± 3.86	5.50 ± 3.55	10.85 ± 5.02
	No	13.00 ± 3.74	6.87 ± 2.36	3.50 ± 2.78	7.38 ± 3.50	13.63 ± 3.20
EGO involvmenent	Yes	10.58 ± 5.38	7.67 ± 3.82	5.83 ± 4.80	6.33 ± 3.17	11.50 ± 5.25
	No	10.41 ± 3.91	6.59 ± 4.25	5.77 ± 3.29	5.73 ± 3.83	11.50 ± 4.61
Stressfull event	Yes	9.78 ± 4.15	7.67 ± 4.50	5.94 ± 3.76	6.72 ± 3.79	11.39 ± 4.68
	No	11.25 ± 4.68	6.19 ± 3.53	5.63 ± 4.00	5.06 ± 3.21	11.63 ± 5.00
Koebner phenomenon	Yes	10.25 ± 4.49	7.40 ± 4.68	7.05 ± 4.19*	5.35 ± 3.18	11.15 ± 4.48
	No	10.79 ± 4.42	6.36 ± 3.08	4.00 ± 2.35	6.79 ± 4.04	12.00 ± 5.28

*Note*: Bold values: the score is significantly higher in subgroup analysis.

Abbreviations: EGO, external genital organs; F, female; M, male; NSV, non segmental vitiligo; SV, segmental vitiligo.

*p < 0.05.

Cyclothymic temperament was positively correlated with DLQI and negatively correlated with age in the vitiligo group (rho = 0.417, *p* = 0.014 and rho = −0.415, *p* = 0.015 respectively) (Table 3). There was no significant correlation between affective temperament scores and disease duration. Positive correlations with cyclothymic and negative correlations with hyperthymic scores were found with the depressive and anxious subscales (Table 3). Depressive and anxious temperaments were positively correlated in vitiligo patients (rho = 0.573, *p* = 0.001). Cyclothymic and irritable subscales were also positively correlated (rho = 0.562, *p* = 0.001).

**TABLE 3:** Correlations between age, disease duration, DLQI and temperament scores in vitiligo patients

		DD	DLQI	Depressive	Cyclothymic	Hyperthymic	Irritable	Anxious
Age	rho	0.055	−**0.344**	−0.058	−**0.415**	0.035	−0.281	−0.329
	p	0.761	0.046	0.744	0.015	0.845	0.107	0.057
	n	33	34	34	34	34	34	34
DD	rho		0.284	0.016	0.065	−0.077	−0.190	−0.032
	p	.	0.110	0.929	0.719	0.669	0.291	0.860
	n		33	33	33	33	33	33
DLQI	rho			0.095	**0.417**	−0.043	0.109	0.283
	p	.	.	0.591	0.014	0.809	0.539	0.104
	n			34	34	34	34	34
Depressive	rho				**0.409**	−**0.588**	0.245	**0.573**
	p	.	.	.	0.016	0.001	0.163	0.001
	n				34	34	34	34
Cyclothymic	rho					−0.116	**0.562**	**0.422**
	p	.	.	.	.	0.514	0.001	0.013
	n					34	34	34
Hyperthymic	rho						0.011	−**0.439**
	p	.	.	.	.	.	0.952	0.009
	n						34	34
Irritable	rho							**0.345**
	p	.	.	.	.	.	.	0.046
	n							34

*Note*: Bold values: the score is significantly higher in subgroup analysis.

Abbreviations: DD, Duration of the disease; DLQI, Dermatology Life Quality Index0; p. n, number of patients; rho, Spearman's rank correlation coefficient (ρ).

## 5 DISCUSSION

This study, the first to investigate affective temperament traits in patients with vitiligo using TEMPS‐A, found that these patients might have a distinct temperament profile. Patients with vitiligo scored higher in anxious and lower in hyperthymic temperaments compared to controls. Cyclothymic temperament was positively correlated with DLQI and negatively correlated with age in the vitiligo group.

Numerous studies have explored the association between psychiatric disorders and vitiligo. Patients with vitiligo have often been described as having anxiety and/or depressive disorders, social phobia, obsessive symptoms, sleeplessness and alexithymia.^20^ However, the affective temperament profile of vitiligo patients has rarely been investigated.

Güler *et al.* evaluated the temperament profile of 27 adult patients with vitiligo using Cloninger's TCI.^22^ The ‘novelty seeking’ score (especially ‘impulsiveness’ sub‐dimension) was significantly lower in patients with vitiligo compared to controls. ‘Novelty Seeking’ dimension quantifies the extent to which a person is impulsive, enthusiastic and easily bored. Similar results were reported by Erfan et al^20^ who investigated the temperament profile of 50 vitiligo patients using TCI and compared them to two groups (alopecia areata and healthy controls). The mean ‘novelty seeking’ dimension score (mainly the ‘extravagance’ and ‘disorderliness’ subdimensions) was significantly lower in vitiligo patients, while mean ‘harm avoidance’ dimension (especially ‘worry and pessimism’ subdimension) was significantly higher. Comparable results were reported in paediatric vitiligo patients.^29^

The links between vitiligo and temperament might be genetic (since both vitiligo and temperament have genetic determinants), and/or explained by different pathophysiological mechanisms, possibly involving neurotransmitters, neurotrophic factors, as well as endocrine (mainly the hypothalamo‐pituitary‐adrenal axis),^30^ and immunological mechanisms (mainly through an association between temperament and the levels of certain pro‐inflammatory cytokines). However, the exact pathological mechanism of the initiation and progression of the disease is not clear.^31^

Studies assessing temperament profile in vitiligo patients have been performed using different instruments making the comparison between results difficult. Nonetheless, these findings could be considered consistent with our results. TCI's ‘Novelty Seeking’ dimension is positively associated with hyperthymic temperament, while ‘harm avoidance’ dimension is strongly positively associated with anxious temperament.^25^ In our study, vitiligo patients had significantly higher anxious and lower hyperthymic temperament scores as compared to controls. The latter is considered a more resilient temperament.^32^, ^33^

Being more likely to have an anxious, and less likely to have a hyperthymic temperament, patients with vitiligo might be less resilient to psychological stressors.^34^ Hence, when exposed to a stressor, patients with vitiligo might be more likely to demonstrate the negative effects of this stressor than those with more adaptive temperamental traits (ref TEMPS‐A pso).

Vitiligo has a major impact on patients' self‐esteem and social interactions and has been associated with psychiatric morbidities such as depression and anxiety.^28^ Several psychological factors may affect the onset and progression of several skin diseases.^35^ The intervention of psychological stress in triggering or aggravating the disease is often reported by patients.^36^ In our study, disease worsening was associated with recent psychological stressors in 52.9% of vitiligo patients. This goes in line with the findings of Silverberg *et al.* who found that 56.6% of patients with vitiligo experienced at least one major life stressor within the 2 years preceding vitiligo onset.^37^ The impact of vitiligo on patient's quality of life as measured using DLQI in our patients was considerable and was correlated with cyclothymic temperament. The higher anxious–depressive traits in female patients was in line with general population studies.^38^

### 5.1 Strengths

To our knowledge, the present study is the first to investigate the affective temperament of vitiligo patients using the TEMPS‐A. Our findings broaden the understanding of the relationship between vitiligo and affective temperament. This work demonstrated that patients with vitiligo may have a distinct temperament profile, perhaps making them more vulnerable to certain medical conditions associated with psychological determinants. A better understanding of the affective traits of vitiligo patients will allow personalising and adapting the treatment, both in the psychological and somatic aspects of this chronic skin disorder.

### 5.2 Limitations

Although an attempt was made to avoid methodological shortcomings, some limitations need to be taken into account. First, this study had a single‐time cross‐sectional design. Second, data were collected from a specific group of fairly well‐educated patients. Patients who could not understand or answer the TEMPS‐A items were excluded. Thus, our findings might not be easily generalised to the whole vitiligo population. Besides, the self‐administered personality test intrinsically relies on the patient's perspective and may have been altered by the patient's purpose to show a better personality state.^27^ In addition, we excluded patients with psychiatric disorders based on the patients' self‐report of psychiatric history. The use of semi‐structured interviews to screen for the most common psychiatric disorders could have helped identify and thus exclude patients with undiagnosed or unreported psychiatric disorders.

In conclusion, this study provides evidence that patients with vitiligo might have a distinct temperament profile characterised by lower hyperthymic and higher anxious traits. These findings also support the idea that the patient's affective temperaments may have some role in the onset of vitiligo and other medical conditions associated with psychological determinants. Such findings should be taken into account when counselling vitiligo patients. Indeed, understanding the temperament traits of vitiligo patients would be essential in establishing a cooperative relationship between patients, dermatologists, and psychiatrists. Moreover, it is possible that a psychotherapy centred on the patient's coping mechanisms with stressful factors, in a subpopulation of patients with vitiligo (with high anxious temperament scores) might improve the patient's overall prognosis and quality of life. Further studies are needed to validate those results in larger sample size and to determine the relationship between affective temperaments, vitiligo, disease triggers, and response to treatment in a longitudinal design.

## AUTHOR CONTRIBUTION

**Noureddine Litaiem**: Data curation (Equal); Methodology (Equal); Supervision (Equal); Writing – review & editing (Equal). **Olfa Charfi**: Conceptualisation (Equal); Methodology (Equal); Writing – original draft (Equal). **Sami Ouanes**: Methodology (Equal); Validation (Equal); Writing – review & editing (Equal). **Soumaya Gara**: Formal analysis (Equal); Writing – review & editing (Equal). **Faten Zegaloui**: Conceptualisation (Equal); Validation (Equal).

## CONFLICT OF INTEREST

The author declares that there is no conflict of interest that could be perceived as prejudicing the impartiality of the research reported.

## ETHICS STATEMENT

Not Applicable.

## Data Availability

Data supporting this study findings are available on Mendeley Data at https://data.mendeley.com/datasets/kj9smj4v9v/2.
